# Key factors affecting ammonium production by an *Azotobacter vinelandii* strain deregulated for biological nitrogen fixation

**DOI:** 10.1186/s12934-020-01362-9

**Published:** 2020-05-19

**Authors:** Mary H. Plunkett, Carolann M. Knutson, Brett M. Barney

**Affiliations:** 1 Department of Bioproducts and Biosystems Engineering, University of Minnesota, 1390 Eckles Avenue, St. Paul, MN 55108-6130 USA; 2 Biotechnology Institute, University of Minnesota, St. Paul, MN 55108 USA

**Keywords:** *Azotobacter vinelandii*, *nifLA*, Ammonium, Nitrogen fixation, Optimization

## Abstract

**Background:**

The obligate aerobe *Azotobacter vinelandii* is a model organism for the study of biological nitrogen fixation (BNF). This bacterium regulates the process of BNF through the two component NifL and NifA system, where NifA acts as an activator, while NifL acts as an anti-activator based on various metabolic signals within the cell. Disruption of the *nifL* component in the *nifLA* operon in a precise manner results in a deregulated phenotype that produces levels of ammonium that far surpass the requirements within the cell, and results in the release of up to 30 mM of ammonium into the growth medium. While many studies have probed the factors affecting growth of *A. vinelandii*, the features important to maximizing this high-ammonium-releasing phenotype have not been fully investigated.

**Results:**

In this work, we report the effect of temperature, medium composition, and oxygen requirements on sustaining and maximizing elevated levels of ammonium production from a nitrogenase deregulated strain. We further investigated several pathways, including ammonium uptake through the transporter AmtB, which could limit yields through energy loss or futile recycling steps. Following optimization, we compared sugar consumption and ammonium production, to attain correlations and energy requirements to drive this process in vivo. Ammonium yields indicate that between 5 and 8% of cellular protein is fully active nitrogenase MoFe protein (NifDK) under these conditions.

**Conclusions:**

These findings provide important process optimization parameters, and illustrate that further improvements to this phenotype can be accomplished by eliminating futile cycles.

## Background

*Azotobacter vinelandii* is a diazotrophic (nitrogen fixing), obligate aerobe studied extensively as a model organism for biological nitrogen fixation (BNF), a complex and energy-intensive process that is stringently regulated by the cell [1–4]. BNF is tightly regulated due to the energetically expensive nature of the reaction which consumes a minimum of 16 mols of ATP and 8 mols of electrons per mol of N_2_ fixed [4, 5]. Primary expression of the genes encoding nitrogenase in *A. vinelandii* is regulated by the *nifLA* operon clustered in a region of the genome located a significant distance from the *nifHDK* genes that encode the catalytic subunits of nitrogenase (NifH and NifDK) [6–8]. Previous evidence suggests that NifA acts as an activator of nitrogenase expression, while NifL acts as an anti-activator, sensing available nitrogen, intracellular redox status, and carbon availability within the cell [9]. Several laboratories have disrupted the *nifL* gene in *A. vinelandii*, resulting in deregulation of nitrogenase expression and subsequent extracellular ammonium accumulation reported to reach up to 30 mM in the supernatant in the late exponential and stationary phases of growth [6, 10–14]. Though much has been elucidated in relation to the nature of deregulation of BNF, there remains aspects of this nitrogen-accumulating phenotype that impede a complete understanding of BNF, and how the cell restructures metabolism during this process.

Our laboratory has reconstructed our own version of the *nifL* deletion resulting in high-ammonium accumulation in the spent medium; *A. vinelandii* strain AZBB163 [11]. This deregulated strain is a powerful tool to probe the overall changes that occur when *A. vinelandii* redirects substantial amounts of energy and resources toward the primary goal of BNF resulting from increased expression of nitrogenase. We recently completed a global transcriptomic study during peak ammonium production in this deregulated strain, which confirmed dramatic increases in the transcription of nitrogenase genes [6], and also provided a glimpse of how the cell regulated multiple other supporting pathways during ammonium accumulation. This study was important, because it provides an opportunity to study a state that is likely transient in typical diazotrophic growth [6, 15].

*Azotobacter vinelandii* has been studied for many decades in relation to the features associated with diazotrophic growth of the wild-type strain [1, 16–18]. Under diazotrophic culture conditions, BNF is balanced and tightly regulated, assuring that the cell produces only as much nitrogen as is required to sustain its own growth. Factors that affect the rate of BNF in wild-type cells are generally indirectly measured through growth rate or using non-native substrates such as acetylene, that result in a terminal product that can be easily quantified [19, 20]. Deregulated strains of nitrogen fixing bacteria such as AZBB163 are reprogrammed to express nitrogenase regardless of internal nitrogen requirements, and could be a valuable tool in determining the specific factors that might limit BNF in this phenotype. Based on the potential applications of these deregulated strains as an alternative for producing high levels of fixed nitrogen, and the opportunity to measure production directly by monitoring levels of ammonium accumulation in spent culture medium, we thought it important to investigate and characterize the different factors that limit production of ammonium in AZBB163. These studies include environmental parameters related to the culture conditions alone and in tandem with further genomic modifications. We investigated potential competing pathways that we hypothesized would become exacerbated under the conditions associated with the high ammonium accumulating phenotype, and might result in energetically costly and futile cycles that inadvertently limit BNF, such as the ammonium importer, AmtB [21, 22]. A description of these parameters and pathways that were targeted, and the findings associated with them, are presented herein, and demonstrate a potential to further improve ammonium production by the strain, and yield additional products that could further enhance the economic potential of pursuing routes to biofertilizer production through BNF [23–25].

## Results

### The effect of varied temperature on ammonium production

**Fig. 1:**
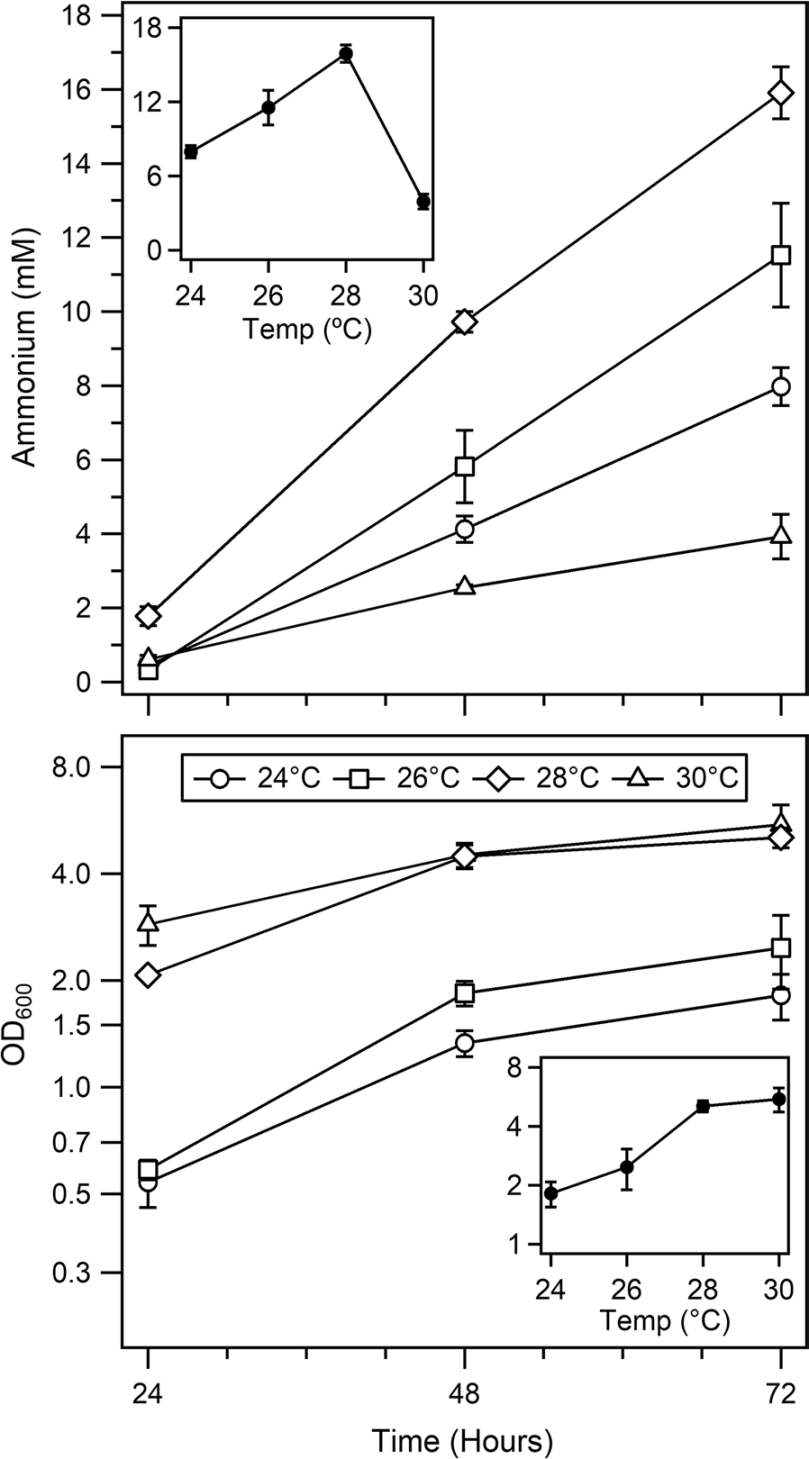
Ammonium production in AZBB163 grown at varied temperatures. Shown are the ammonium production (top) and OD_600_ (bottom) for AZBB163 grown at 24 °C, 26 °C, 28 °C, and 30 °C. Cultures were grown in Burk’s medium and were shaken at 180 rpm in 125 mL Erlenmeyer flasks with 60 mL of medium. Bottom graph optical density plotted on a log_2_-based scale (y-axis). Data points indicate averages, while error bars represent standard deviation (N = 4). Insets show the yield obtained at 72 h (top) and the OD_600_ obtained at 72 h (bottom) versus temperature

During initial experiments described here that test specific parameters or genetic manipulations, care was taken to only alter one parameter at a time, and test against the base *A. vinelandii* AZBB163 strain (hereafter referred to as simply AZBB163), which yields high quantities of ammonium. This was done so that differences between individual experiments could be compared to one another and to previous reports [6, 10–12]. In later experiments, multiple growth parameters and genetic modifications were combined to test specific hypotheses. The AZBB163 strain was constructed to replicate the reports by Bali et al. and Brewin et al. [10, 12]. The first parameter tested was temperature. Ammonium levels achieved by AZBB163 cultures grown at different temperatures varied significantly, with 28 °C resulting in the highest ammonium concentrations per culture, and 26 °C resulting in the highest ammonium per culture density (Fig. 1). OD_600_ values obtained during the culture were not significantly different between the samples grown at 24 °C and 26 °C, but 28 °C and 30 °C resulted in cultures that achieved a slightly elevated OD_600_. Interestingly, while total ammonium levels increased steadily from 24 to 28 °C, 30 °C resulted in a steep decrease in the rate of ammonium accumulation, indicating that slight increases could have profound effects on the culture and a negative effect above a certain threshold for ammonium yields.

### The effect of culture volume on ammonium production

**Fig. 2:**
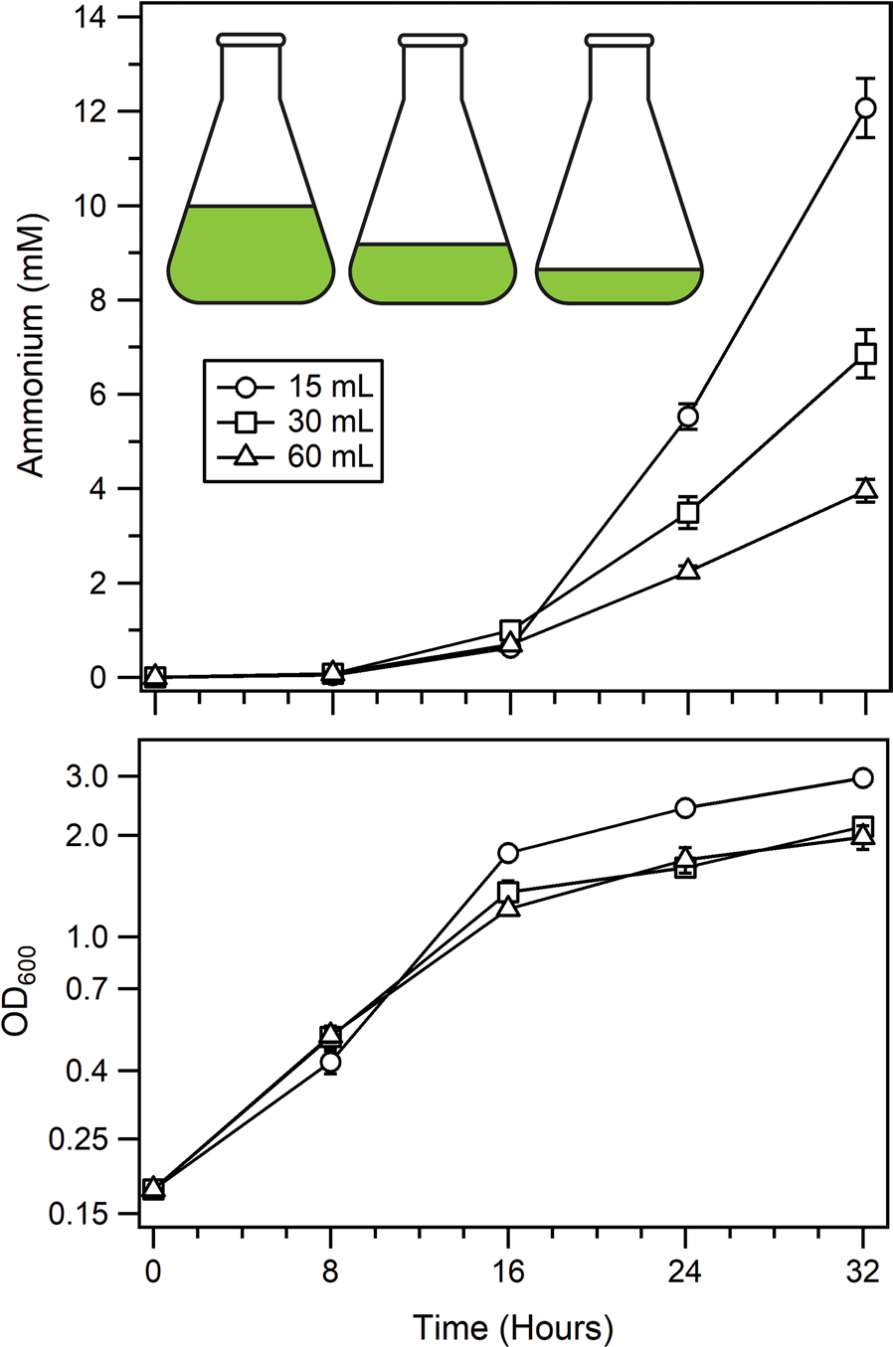
Ammonium production in AZBB163 in cultures with varied volumes. Shown are the ammonium production (top) and OD_600_ (bottom) results from growing AZBB163 as 15 mL, 30 mL and 60 mL cultures. Cultures were grown in Burk’s (B) medium with 20 g/L starting sucrose concentration at 26 °C and shaken at 180 rpm in 125 mL Erlenmeyer flasks. Bottom graph optical density plotted on a log_2_-based scale (y-axis). Data points indicate averages, while error bars represent standard deviation (N = 5)

*Azotobacter vinelandii* is an obligate aerobe, and given the high ATP demand related to excess nitrogen fixation in AZBB163, we were curious whether oxygen availability may limit growth or hinder nitrogen fixation. A simple approach to increase oxygen availability in batch culture is to decrease the total culture volume within the flask while keeping the flask size constant, providing a greater proportion of exposed surface area to atmosphere versus the volume of culture and more vigorous mixing. Ammonium production was tested in cultures with volumes of 15 mL, 30 mL, and 60 mL each in 125 mL Erlenmeyer flasks (Fig. 2). All three volumes yielded similar concentrations of ammonium up to 16 h. However, between 16 and 32 h, the cultures significantly deviated with respect to ammonium concentration, with the cultures containing 15 mL of medium producing the highest concentrations of ammonium, followed by the 30 mL cultures, and finally the 60 mL cultures. All conditions resulted in similar culture densities up to 8 h of growth, after which the 15 mL cultures grew more densely, while the 30 mL and 60 mL cultures maintained a lower OD_600_. While variation associated with evaporation rates within cultures of smaller volumes was a concern, analysis revealed that there was a negligible decrease in culture volume over the duration of the experiment due to evaporation, indicating that the differences in ammonium levels were independent of losses in culture volume based on the experimental design.

Alternative experiments that employed baffled flasks in an effort to further increase the aeration resulted in a sharp drop in levels of ammonium achieved, and increased foaming and cellular aggregation, while efforts to increase aeration further (described below), were not detrimental to cell dispersion and ammonium accumulation. These results indicate that alternative approaches to increase oxygen availability should be approached with caution.

### Disruption of a futile cycle associated with ammonium import

**Fig. 3:**
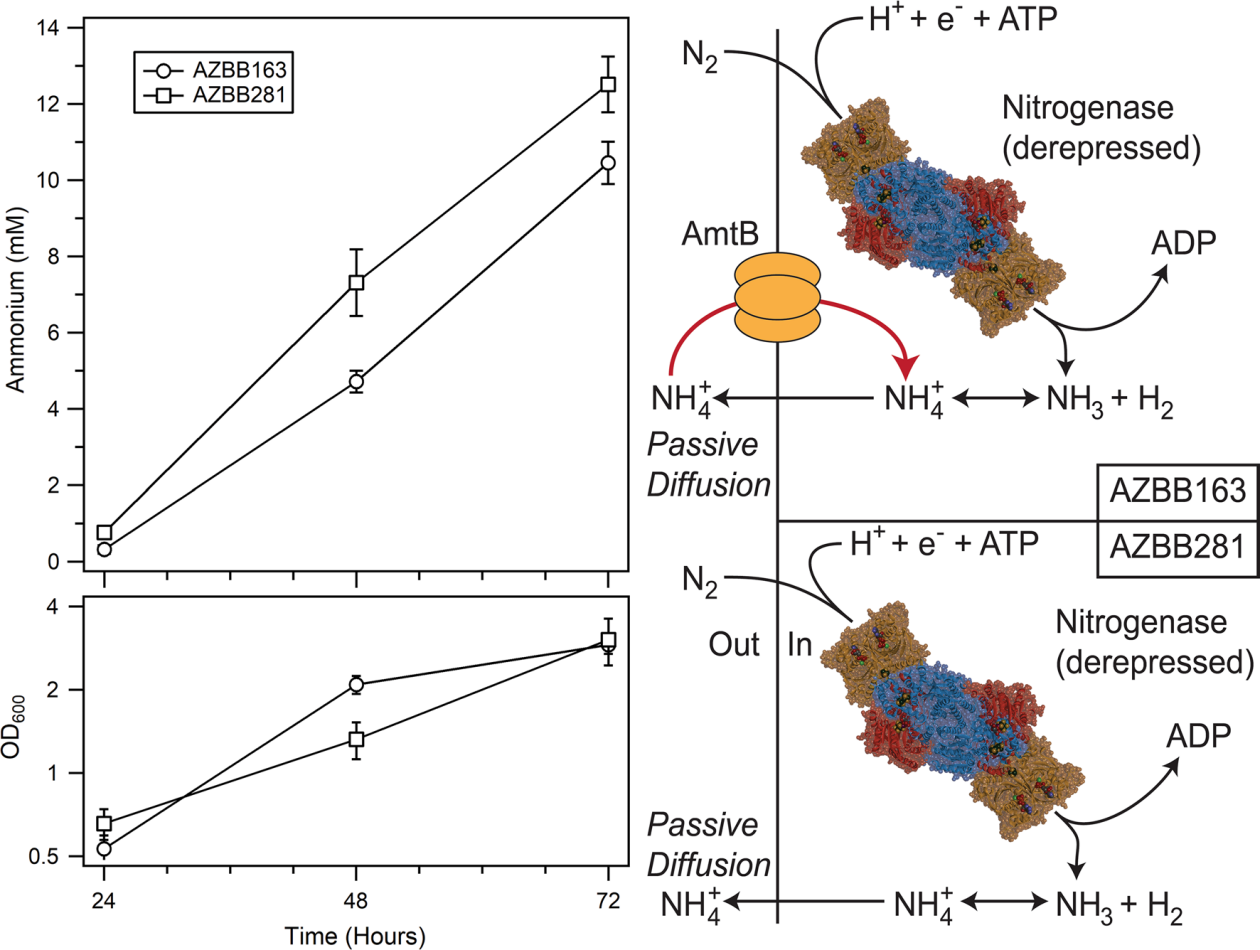
Ammonium production in a strain lacking the ammonium transporter AmtB. Shown are the ammonium (top left) and OD_600_ (bottom left) results of growing AZBB163 and AZBB281 (AZBB163 derivative containing the *amtB* deletion) in Burk’s (B) medium. Shown on the right is a graphical representation of the effects of deleting *amtB* within a strain of *Azotobacter* deregulated for nitrogen fixation. Cultures were grown at 26 °C and shaken at 180 rpm. Bottom graph optical density plotted on a log_2_-based scale (y-axis). Data points indicate averages, while the error bars represent standard deviation. N = 4

The ammonium transporter AmtB is constitutively expressed in *A. vinelandii*, regardless of extracellular ammonium levels [6]. In a strain such as AZBB163 that has been deregulated for nitrogen fixation, the active transport of ammonium back into the cell would represent a potentially futile and energy wasting cycle (Fig. 3). We hypothesized that if the *amtB* gene were deleted, it might result in a higher rate of ammonium accumulation within the supernatant. Using a strain from a previous report [11, 26], the *nifL* deletion that results in extracellular ammonium accumulation was added to a strain containing the *amtB* deletion, creating *A. vinelandii* strain AZBB281. When evaluated for ammonium production, our results demonstrated that AZBB281 accumulated ammonium in the supernatant at a faster rate as compared to AZBB163 at both day 2 and day 3 (Fig. 3), illustrating that this process may represent an energetically wasteful process.

### The effect of increased metals and sulfur concentrations on ammonium production

**Fig. 4:**
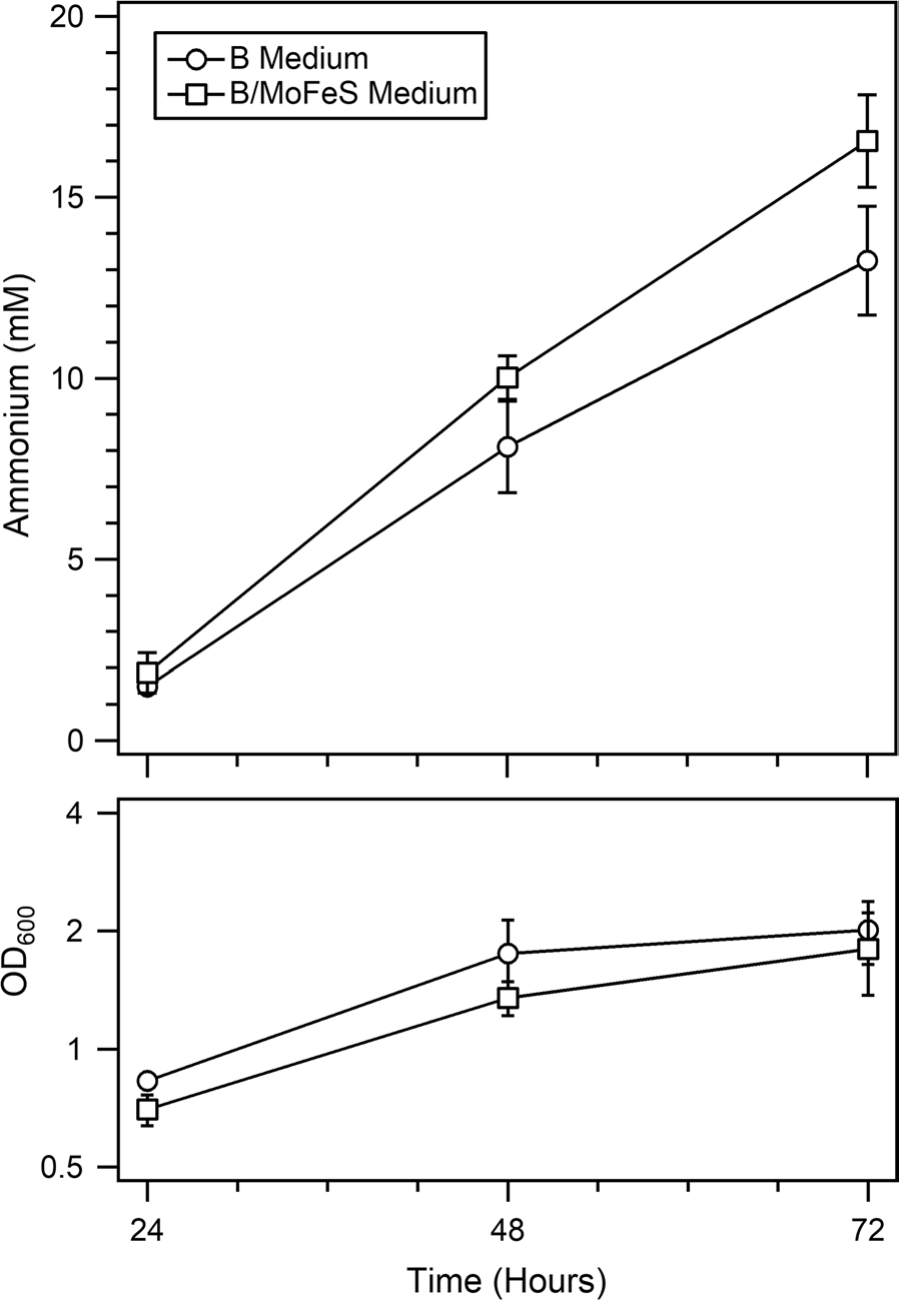
Ammonium production in AZBB163 grown on Burk's medium versus B/MoFeS medium. Shown are the ammonium production (top) and OD_600_ (bottom) results from growing AZBB163 in both standard Burk’s (B) medium and B/MoFeS medium (supplemented with nitrogenase cofactor-related elements containing twofold increase in iron (Fe), fivefold increase in molybdenum (Mo), and threefold increase in sulfur (S) versus B medium). Cultures were grown at 26 °C and shaken at 180 rpm, and were grown as 60 mL cultures in 125 mL Erlenmeyer flasks. Bottom graph optical density plotted on a log_2_-based scale (y-axis). Data points indicate averages, while error bars represent standard deviation (N = 5)

In an effort to ameliorate the potential limitations of essential elements required for cofactor assembly related to nitrogenase, a supplemented medium was developed via a series of experiments testing increases in molybdenum, iron and sulfur (B/MoFeS); elements essential to metal cluster assembly for nitrogenase component proteins [4]. These preliminary experiments established levels that increased availability of these elements without inducing a detrimental response due to potential toxicity. The adapted medium (B/MoFeS) balanced an increase in ammonium production with a minimal increase in the concentration of each of these three elements, resulting in the final optimized medium containing sulfur increased threefold, molybdenum fivefold, and iron twofold as compared to standard B medium. AZBB163 was cultured in this medium optimized for high levels of nitrogenase expression (Fig. 4). The results demonstrate a statistically significant ~ 25% increase in the amount of ammonium produced on days 2 and 3 of growth, while there was a significant decrease in the OD_600_ on days 1 and 2 in the presence of optimized cofactor elements (p ≤ 0.05).

### Sucrose consumption and limitation in AZBB163

**Fig. 5:**
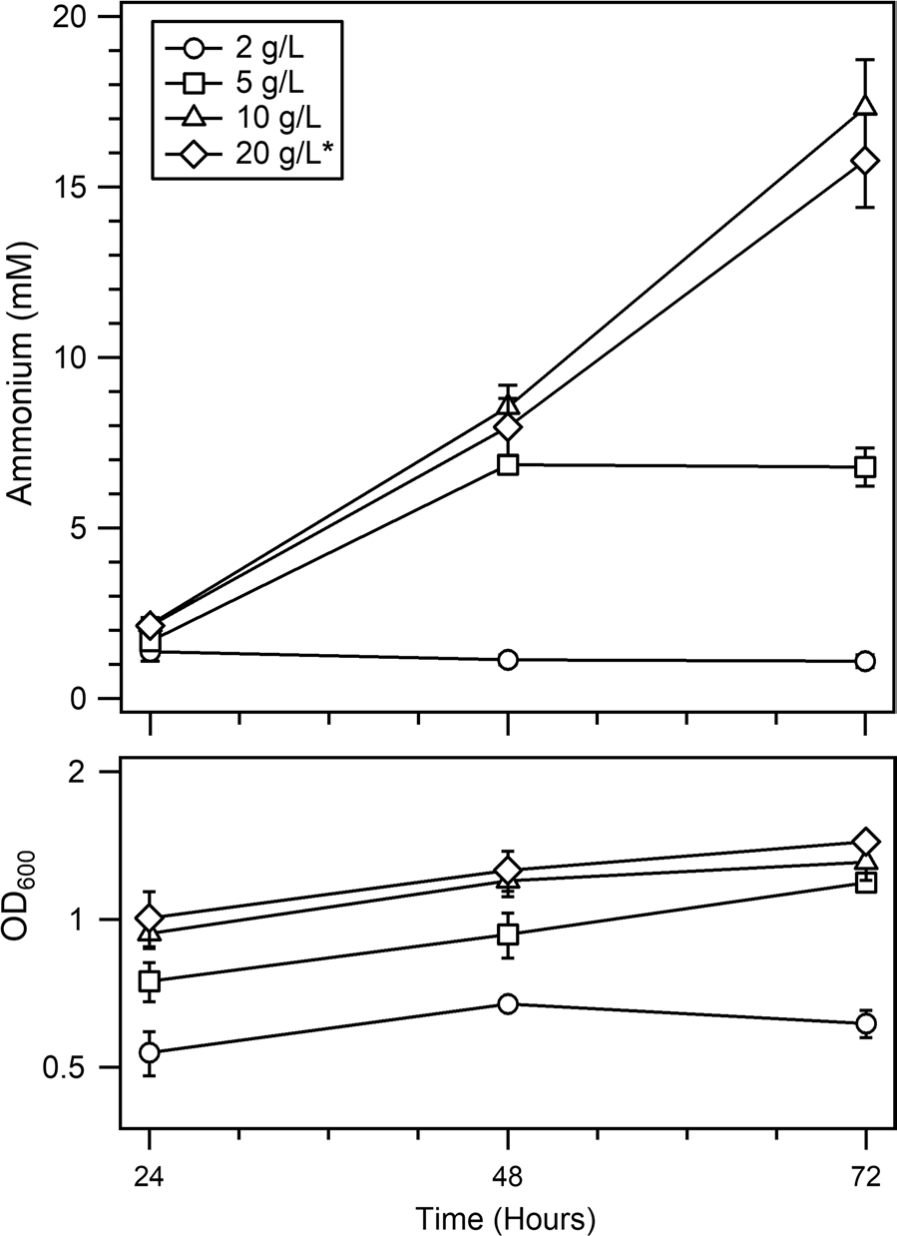
Ammonium production in AZBB163 grown in B/MoFeS medium with varied concentrations of sucrose. Shown are the ammonium production (top) and OD_600_ (bottom) results from growing AZBB163 in B/MoFeS medium (Burk’s medium supplemented with increased cofactor-related elements) with varied sucrose concentrations, with the standard concentration of sucrose (20 g/L) indicated with an (*). Cultures were grown at 26 °C and shaken at 180 rpm, and were grown as 60 mL cultures in 125 mL Erlenmeyer flasks. Bottom graph optical density plotted on a log_2_-based scale (y-axis). Data points indicate averages, while error bars represent standard deviation (N = 4)

Our next aim in these experiments was to quantify yields of ammonium in relation to growth substrate utilization. These initial experiments utilized the improved medium containing elevated cofactor elements described above, but remained focused on strain AZBB163. As a first step in this understanding, we looked at rates of ammonium accumulation and substrate consumption over a period of 3 days. Varied concentrations of sucrose in B/MoFeS medium were tested in growths of AZBB163, and ammonium levels were analyzed to determine when cultures limited in sucrose would plateau in ammonium production (Fig. 5). B/MoFeS medium was utilized to ensure that sucrose, and not a shortage in the elements related to cofactor assembly, was the limiting factor for growth and ammonium production. The selection of this medium was partially based on limitations in maintaining soluble metals when sugar levels are lower than what is found in standard B medium. Cultures containing 2 g/L became limited for sucrose after 1 day of growth, whereas cultures grown in 5 g/L of sucrose plateaued in ammonium production after 2 days of growth. The cultures containing 10 g/L of sucrose and 20 g/L sucrose (the typical concentration in B medium) generated very similar levels of extracellular ammonium and culture density on all 3 days of the experiment.

### Ammonium yields versus sucrose consumption under optimized conditions

**Fig. 6:**
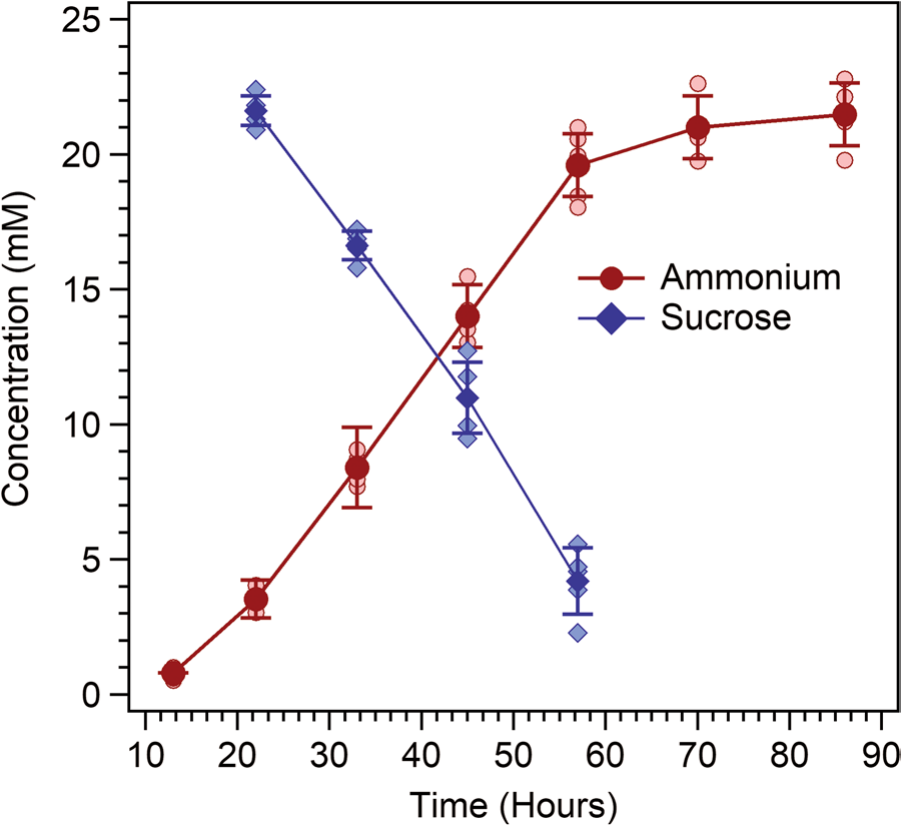
Ammonium production and sucrose consumption in AZBB281 grown in sugar-limited (~ 29 mM Sucrose) B/MoFeS medium. Shown are the ammonium production and sucrose consumption results obtained when growing AZBB281 in Burk’s medium supplemented with cofactor-related elements (B/MoFeS medium) with 10 g/L of starting sucrose concentration (1/2 of sucrose concentration in typical B medium). Cultures were grown at 26 °C and shaken at 200 rpm in 125 mL Erlenmeyer flasks with 60 mL of medium. Data points indicate averages, while error bars represent standard deviation (N = 5). All data points (lighter shade) are included for reference

One question arising from the results described above related to the trade off in ammonium produced versus sucrose consumed. Results provided in Fig. 5 revealed that ammonium levels approaching 20 mM were achieved with half of the sucrose that is typically required to reach 25–30 mM ammonium (see below). In order to more accurately profile the rate of sucrose consumption under optimized conditions, initial sucrose concentrations in B/MoFeS medium were lowered to 10 g/L and cultures were grown beyond the point of sucrose depletion using strain AZBB281, the strain lacking the futile cycle for ammonium recycle (Fig. 6). After 3 days, nearly all sucrose was consumed from the medium, and increases in ammonium concentration had plateaued.

The highest rates of ammonium production and sucrose consumption occurred between 22 and 57 h. The ratio of mmols of ammonium produced per mmol of sucrose consumed was 0.93 ± 0.07 during this window of time. Under these conditions, the cultures were inoculated into medium containing approximately 29 mM of sucrose and ceased accumulation of ammonium at concentrations of 21.5 ± 1.1 mM ammonium, making the net conversion approximately 1 mol ammonium produced per 1.35 mol sucrose consumed.

### Maximizing rates of ammonium production

**Fig. 7:**
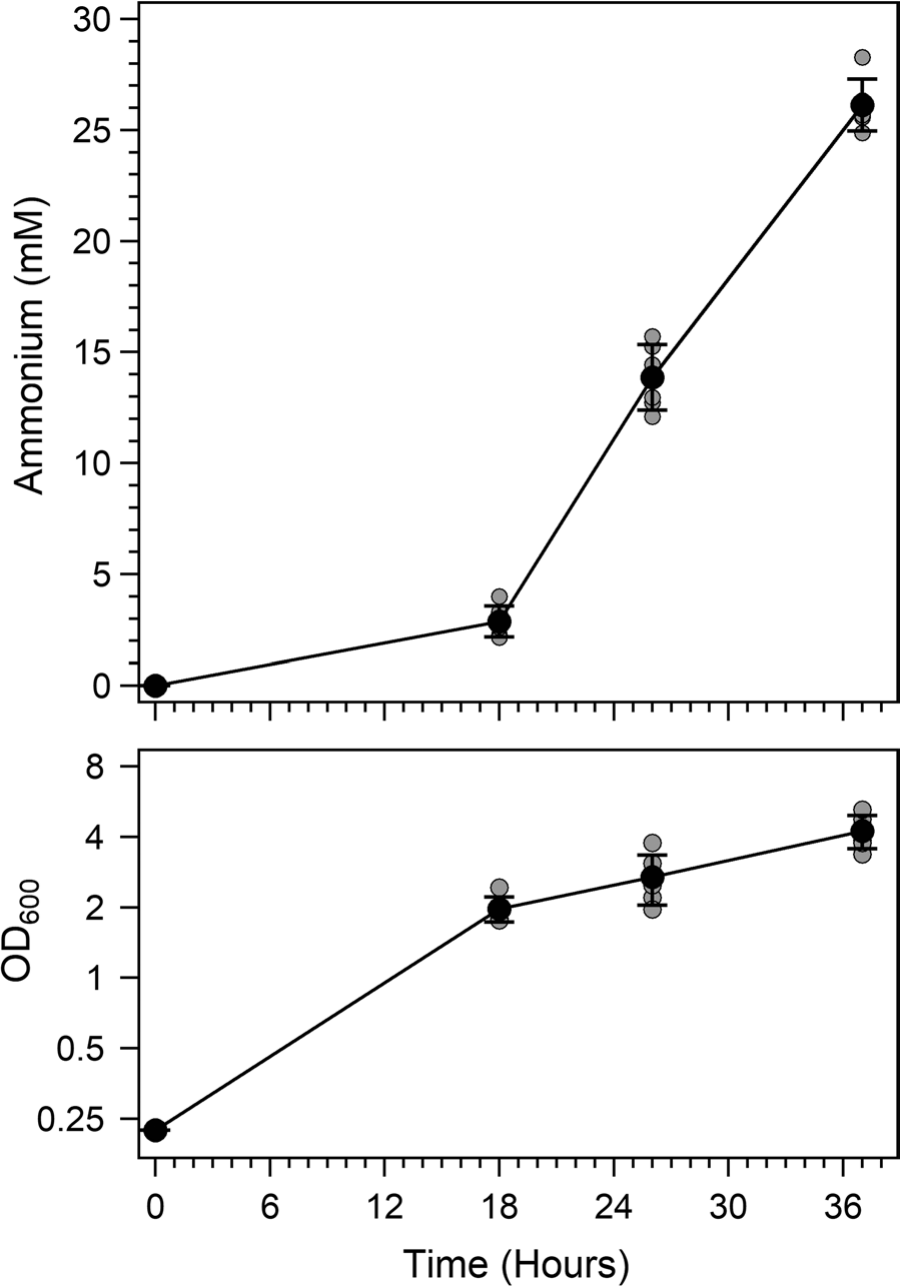
Ammonium production in AZBB281 grown under optimized conditions. Shown are the ammonium production (top) and OD_600_ (bottom) of AZBB281 grown under conditions found to produce the most ammonium per unit time. Cultures were grown at 28 °C, with 15 mL culture volumes, in Burk’s medium supplemented with additional nitrogenase cofactor-related elements (B/MoFeS medium), and shaken at 300 rpm. Bottom graph optical density plotted on a log_2_-based scale (y-axis). Data points indicate averages, while error bars represent standard deviation (N = 5). All data points (lighter shade) are included for reference

As a final experiment, we sought to incorporate each of the identified optimal conditions with the optimized genetic construct to compare the improvements that could be achieved (Fig. 7). Our goal in this experiment differed from those described above. In this experiment, we sought to determine the highest rate of ammonium accumulation in the extracellular space, regardless of the sucrose required to achieve these rates. Strain AZBB281 was grown at 28 °C, in 15 mL of B/MoFeS medium. These combined conditions yielded the highest rate of ammonium production over time from any of these experiments. The experiment was run at 300 rpm until it reached an ammonium level of greater than 25 mM. During the 8-h period between 18 and 26 h for the experiment, cultures achieved rates of approximately 1.37 mM of extracellular ammonium accumulated per hour. This value is equivalent to nearly 23 μM per minute (23 nmol/min per mL). Protein levels between 18 and 26 h averaged about 300 μg/mL, indicating an ammonium yield of about 75 nmol/min/(mg total cellular protein). To yield 23 nmol/min of ammonium with purified nitrogenase enzyme requires approximately 38 μg of MoFe protein (NifDK) with an excess of Fe protein (NifH) [27, 28]. Based on these calculations, 8–13% of the cellular protein is required as MoFe protein (NifDK) functioning at optimal rates with full activity [27, 29]. Achieving these rates in vitro required 10 molar equivalents of Fe protein to MoFe protein [28], while others have reported Fe:MoFe protein ratios of ~ 2:1 within *A. vinelandii* wild-type cells during maximum in vivo nitrogenase activity [30]. These protein levels agree with previous findings related to protein expression profiles for these BNF deregulated strains [6].

## Discussion

Optimizing growth parameters to maximize the production of desired products is a simple, yet robust approach that could compliment what more difficult genetic manipulations are able to achieve. In this work, both culture conditions and further genetic manipulations were employed independently and in combination to yield further increases in the accumulation of ammonium in *A. vinelandii* strains partially deregulated for BNF. These studies provide insight into the limiting factors that *A. vinelandii* may face when deregulated for BNF and producing copious quantities of extracellular ammonium. The growth parameters investigated in this study included oxygen limitation, temperature, and availability of the Mo, Fe and S for the biosynthesis of cofactors essential for nitrogenase. One aspect that became evident as a result of these findings is that there is a balancing act that must be taken into consideration when seeking to optimize conditions to maximize either yields or rate of ammonium production through BNF in *A. vinelandii*.

We approached the questions regarding the role of individual growth parameters through an incremental strategy, altering only one parameter in each experiment, and retaining the common growth parameters for all other aspects of growth. Modifying the growth temperature had a profound and intriguing effect on the amount of ammonium produced. The highest temperature tested (30 °C) was found to be detrimental to ammonium accumulation in the extracellular space versus slight decreases in culture temperature, and resulted in a trade-off in limiting ammonium while increasing culture density (Fig. 1). Growing cultures at a slightly lower temperature of 28 °C resulted in the highest rates of ammonium accumulation in the medium. Lowering the temperature to 26 °C resulted in similarly high ammonium levels, but lowered culture density, and growth at 24 °C gave similarly lowered culture density, but lower levels of ammonium, while still achieving higher levels of ammonium than cultures grown at 30 °C. Typical growth temperatures for culturing *A. vinelandii* are often cited as 30 °C in the primary literature [31–34], and 30 °C is the suggested growth parameters described by the American Type Cultures Collection (ATCC).

This suggested temperature of 30 °C for culturing *A. vinelandii* differs from optimal conditions for respiration described by Lineweaver, Burk and Horner more than 80 years ago, who found that *A. vinelandii* respires at increasingly higher rates up to 35 °C, after which point respiration rates decrease [1]. The effect of temperature on nitrogen fixation in various bacteria has been studied previously by probing a wide range of temperatures using the alternative substrate acetylene, and found optimal rates of activity at 35 °C for *Azotobacter chroococcum* [35]. Additional studies have looked at the regulation of nitrogenase related gene expression at different temperatures in *A. vinelandii* [36, 37]. In general, the activity of isolated nitrogenase enzymes tends to increase with steadily higher temperatures through 45 °C [38, 39]. Most species of *Azotobacter* produce only as much ammonium as is necessary to sustain their own growth. Since many assays of nitrogenase activity done in vivo actually use the substrate acetylene, which has characteristics that differentiate it from N_2_, the strain used in this study provides a unique means of testing the effect of temperature on BNF in a strain specifically deregulated for this process. This optimal temperature for ammonium production contrasts with prior reports. This is not completely unexpected, as the metabolism within this strain has been altered dramatically to achieve this ammonium production phenotype [6, 10–12, 29].

The shift in cell density that occurred between 26 and 28 °C was also unexpected (Fig. 1). We previously reported differences in final cell densities that were obtained between the AZBB163 and the wild-type strain [6]. The stark change that occurred in AZBB163 during this rather narrow temperature range could result from several different sources, including a shift of metabolism that favors polyhydroxybutyrate production or a limitation of other key intracellular metabolites, which both fell outside the scope of this current study. The selection of an optimal temperature with AZBB163 and similar strains derived from AZBB163 is thus dependent on the goals of the experiment. If optimal rates of ammonium accumulation were the goal, then 28 °C would be ideal. However, in the majority of our experiments, we aimed for high ammonium yields from a minimal mass of cells, and in those experiments, we thus selected 26 °C.

Increased cell density should translate to increased competition for available oxygen. It is well established that *A. vinelandii* expends a great deal of energy attempting to carefully modulate internal oxygen levels [1, 40]. Since oxygen solubility in water is actually quite low versus many other gases, and *A. vinelandii* is known to have one of the highest respiratory rates reported for bacteria [1, 18], it is quite possible that oxygen availability could limit BNF and the excess ammonium production that results in the ammonium-releasing phenotype that is associated with AZBB163. In contrast, nitrogenase is an oxygen-sensitive enzyme, and elevated levels of oxygen near the active site of nitrogenase could irreversibly damage this enzyme. This conflicting nature of these two highly dependent processes are at odds with one another. Thus, the AZBB163 strain offered a unique opportunity to determine how well the cell protects these elevated levels of nitrogenase under elevated expression conditions and whether the strain was in fact oxygen limited as a result.

Outside of using a fermenter or other forced aeration device, there are several manners to improve oxygen delivery to batch-grown flask cultures. One approach would be to increase the fraction of oxygen within the atmosphere. However, since *A. vinelandii* also needs the nitrogen from the atmosphere in these experiments to support BNF, such an approach was viewed as self-sabotaging. We thus selected only natural atmospheric compositions of oxygen and nitrogen gases. The addition of baffles to the Erlenmeyer flasks used in these studies was also viewed as a favorable method of introducing more oxygen, but resulted in significant foaming and aggregation of the cells, accompanied by a dramatic drop in ammonium production. Reduction of culture volume, while keeping the flask size constant, is a classical way to provide more oxygen to batch grown cultures by increasing the surface area exposed to atmosphere, resulting in increased aeration [41]. The approach of lowering the volume of culture while maintaining the same size of Erlenmeyer flask clearly demonstrated that ammonium production could be steadily and significantly increased by lowering the ratio of volume versus the available surface area exposed to the standard oxygen atmosphere (Fig. 2). This illustrated that the culture was still oxygen limited under our initial conditions, including those we have reported previously [6, 11], and that the strain is well adapted to protect the nitrogenase from any detrimental effects associated with the oxygen, even with the elevated expression of nitrogenase that accompanies this phenotype [6, 12].

In a previous study investigating the changes in global transcription that occur within AZBB163 [6], we determined that molybdenum availability limited ammonium accumulation in AZBB163. The production of the full complement of metal clusters in each full Mo-based nitrogenase complex (NifDK + 2 NifH) requires 38 Fe and 2 Mo, but also requires 40 S atoms. Each cluster is bound to the enzyme through one or more cysteine ligands. This would also increase the demand for S. Based on the elevated levels of expression of both NifDK and NifH that were found from our prior analysis [6], and the relatively low concentration of sulfate included in the general recipe for Burk’s medium [42], we hypothesized that sulfur might also limit ammonium production in AZBB163. Experiments revealed that elevating the levels of both Mo, Fe and S, independently and in combination, resulted in a slight improvement in ammonium accumulation. Based on these observations, a new medium was formulated that increased levels of Mo by 5×, Fe by 2× and S by 3×. The optimized medium resulted in an increase in ammonium accumulation as compared to standard Burk’s medium (Fig. 4). Though standard Burk’s medium provides sufficient levels of the microelements required to maintain the diazotrophic phenotype for the wild-type strain, the elevated cofactor requirements of AZBB163 to sustain high levels of extracellular ammonium production necessitates an increase in sulfur. Our prior studies with AZBB163 indicated a limitation due to metal depletion by the cell, which manifested as an increase in transcription for siderophore peptides [6, 43], but diminished upon addition of excess metals. These results thus indicate that all three micronutrients involved in the biosynthesis of nitrogenase cofactors should be considered when optimizing BNF through AZBB163 and related strains.

Prior efforts revealed that elimination of the gene *amtB*, which codes for an ammonium transporter, results in the release of ammonium to the extracellular culture medium that is sufficient to support the co-culture of algae [11]. AmtB is thought to serve a role in *A. vinelandii* to shuttle ammonium into the cell by linking this transport to the activity of glutamine synthetase, resulting in the hydrolysis of ATP, which also serves a vital role in BNF [4]. It has also been shown that ammonium and methylamine transport in *A. vinelandii* is dependent on membrane potential [44, 45]. For this reason, any strains manipulated to produce elevated levels of extracellular ammonium are potentially wasting ATP or energy associated with maintaining the membrane potential in an effort to recycle that ammonium back into the cell in a process that should be futile and wasteful. Additionally, since this process is not directly coupled to BNF, it could result in a much higher waste of energy than what would be expected for deletion of the hydrogenase, especially as levels of ammonium accumulate outside of the cell. Contrary to what was found when deleting the hydrogenases [29, 46], the deletion of *amtB* did result in an improvement in the rate of ammonium accumulation (Fig. 3), indicating that this process is likely wasting unnecessary energy and limiting ammonium accumulation.

Our final experiments pursued here combined the collective understanding of the physical parameters and genetic modifications to determine both maximum yields and maximal rates of ammonium generated through AZBB281. This strain contains both the *nifL* disruption that results in deregulation of nitrogen fixation along with the *amtB* deletion that disrupts any futile attempts to transport extracellular ammonium back into the cell. Strains were grown under two sets of conditions. The first approach sought to maximize yield of ammonium per mol of sucrose (Fig. 6). In the second approach (Fig. 7), the goal was to determine maximum rates of yielded per unit time. Our experiment was performed at 300 rpm and 28 °C in the improved B/MoFeS medium in 125 mL Erlenmeyer flasks containing 15 mL of media, which resulted in rates of extracellular ammonium accumulation per hour that surpassed any of the results found in any of the prior experiments (Figs. 1, 2, 3, 4, 5 and 6). In these experiments, rates of extracellular ammonium accumulation surpassed 1 mM per hour, and increased at a relatively linear rate between hours 18 and 37. In these experiments, we provided the standard quantities of sucrose (~ 58 mM) to the medium, and achieved ammonium levels averaging 26 mM at 37 h. This increased rate of ammonium accumulation came at a cost of higher sucrose consumption, as nearly all of the sucrose was consumed by hour 37 (~ 90%). This indicates an ammonium yield of about 0.5 mM ammonium per mole of sucrose, which is lower than what we found with lower quantities of sucrose when aeration and rate of ammonium accumulation was not maximized (Fig. 6). These results highlight the trade-off that is associated with higher rates versus required energy in the form of the sugar substrate, and the maximum concentrations of ammonium that the strain could obtain in liquid culture.

In most of our efforts to test maximal yields of ammonium, the strains are generally able to achieve levels approaching 25–30 mM ammonium, when provided sufficient levels of sucrose. This 30 mM maximum range appears to represent a proverbial “brick wall,” as ammonium levels greater than this value were difficult to obtain, even when sufficient sucrose levels remained in the culture. Thus, slowing the rate of oxygen delivery, and as a result, ammonium accumulation, required ~ 1.4 mol of sucrose per mol of ammonium (Fig. 6), while increasing oxygen delivery to decrease the amount of time required to accumulate slightly elevated levels of ammonium required ~ 2.3 mols of sucrose per mol of ammonium (Fig. 7).

## Conclusions

This report demonstrates potential levels of ammonium yielded under our current best-case scenario for ammonium accumulation by an aerobic organism. It further demonstrates that transport processes recycling ammonium, such as those linked to AmtB, may actually hinder ammonium production rates as a result of futile cycles for a deregulated strain. Yields of ammonium per mole of sucrose illustrate that tradeoffs exist between maximizing rates of ammonium production versus optimizing total yields per mole of sucrose. As a result of these new constructs, an alternative full cell assay is now possible that eliminates the need to study nitrogenase activity through a surrogate such as acetylene, and as a result, we demonstrate that temperatures long believed to be optimal for growth of *A. vinelandii* are actually detrimental to maximizing ammonium production in a BNF deregulated strain.

## Methods

### Genetic constructs, gene deletion and confirmation

*Azotobacter vinelandii* DJ (ATCC BAA-1303) was obtained from Dennis Dean (Virginia Tech) and cultured on Burk’s medium [42]. *Escherichia coli* JM109 was acquired from New England Biolabs (Ipswich, MA) and utilized to construct the plasmids described. Plasmids used to construct final vectors for gene deletions in *A. vinelandii* are described in Table 1. Primers used for confirmation of genetic constructs are described in Table 2. Detailed methods for the transformation of *A. vinelandii* have been described previously [26, 42]. Transformation of *A. vinelandii* DJ in order to modify target genes utilizing double homologous recombination with the *pyrF* counter-selection technique were implemented as described previously [11, 26]. Details for strains constructed for use in this study are provided in Table 3. Methods describing the construction of strains AZBB106, and AZBB163 have been described previously [11, 29].

**Table 1:** Key plasmid constructs utilized in this work

Plasmid^a^	Relevant gene(s) cloned or plasmids manipulated	Parent vector	Source or references
pPCRNH3-44	Incorporated C to T mutation into kanamycin cassette region to create Nif^+^ phenotypes with high ammonium production	pPCRNH3-43	[6]
pPCRPYRF1	Cloned *pyrF* and flanking regions from *A. vinelandii*	pBB053	[26]

^a^The sequences of all plasmids in this study are available upon request

**Table 2:** Key primers used in this study

Primer	Sequence 5′-3′	Purpose
BBP950	GAGCACACCCATCACGGTCAGAG	*nifLA* modification confirmation
BBP1322	GATCTCCATCGACTCGATCTTGTCCAGGGTGAAC	*nifLA* modification confirmation
BBP2006	CACGTGCCAGGAATTCCTCCATG	*amtB* gene deletion confirmation
BBP2007	CTGTGGACGATGGCCAGGGACATGGATC	*amtB* gene deletion confirmation

**Table 3:** Key strains constructed and utilized in this study

*A. vinelandii* strain	Genetic features	Plasmid utilized	Parent strain
DJ (ATCC BAA-1303)	Wild-type *A. vinelandii* with diminished alginate production resulting in ease of transformation	None	None
AZBB109	∆*amtB*	pPCRPYRF1^a^	AZBB106^a^
AZBB163^b^*	*nifL::*Kan^R^(pPCRNH3-43). Was grown in medium without nitrogen source, and mutated to a nif+ phenotype that also produces high levels of extracellular ammonia	pPCRNH3-43, spontaneous mutation	AZBB150^b^
AZBB281*	∆*amtB, nifL*::Kan^R^(pPCRNH3-44)	pPCRNH3-44^c^	AZBB109

Kan^R^ Kanamycin resistance

Strains indicated by an asterisk (*) are completed and were used in ammonium production experiments

^a^ As described previously [26]

^b^ As described previously [11]

^c^As described previously [6]

### Growth of cells and B/MoFeS medium development

*Azotobacter vinelandii* strains were grown in base/acid washed 125 mL Erlenmeyer flasks containing 60 mL of medium unless otherwise stated. Experiments measuring the effect of increased aeration were performed by inoculating a larger volume of sterile medium, and then adding aliquots of different volumes of medium into sterile flasks of the same size (125 mL). All experiments were performed with at least three replicates. Burk’s/Na_2_MoO_4_, FeSO_4_, Na_2_SO_4_ (B/MoFeS) medium was developed using standard Burk’s (B) medium recipes [42] supplemented with 2× iron (18 µM increased to 36 µM as FeSO_4_), 5× molybdenum (1 µM increased to 5 µM as Na_2_MoO_4_), and 3× sulfur (0.8 mM increased to 2.4 mM as Na_2_SO_4_) as determined through a series of experiments testing ranges of these elements. In each experiment, cultures were inoculated with approximately equal initial concentrations of cells (OD_600_ ~ 0.15–0.25) within each particular experiment from cells scraped off B plates containing appropriate antibiotics grown for 2 days at 30 °C. Cultures were grown at 26 °C with agitation at 180 rpm unless otherwise noted. The temperature of 26 °C was chosen as a standard due to the balance of ammonium produced per culture optical density, which was maximal at this temperature. Supernatant was collected by centrifugation (> 12,000*g* for 1 min) of samples at the indicted time points.

#### Ammonium quantification

Assays for ammonium quantification used the colorimetric o-phthalaldehyde method as described previously utilizing a Cary 50 Bio Spectrophotometer measuring absorbance at 412 nm [6].

#### Sucrose quantification

For experiments measuring the levels of sucrose in the medium, the Sigma-Aldrich kit (Part No: MAK013-1KT) was used as directed by the manufacturer.

#### Protein quantification

To quantify the total amount of starting protein, isolated cell pellets were suspended in 1 mL of water and sonicated for 60 s (Misonix LX-2000, Qsonica, Newtown, CT) inside of a 2.0 mL Eppendorf tube, and centrifuged at 13,000*g* for 5 min to remove cell debris. Cell lysate was then added to 1.0 mL of Coomassie Plus (Bradford) assay reagent (Pierce, Rockford, IL), mixed by pipetting and incubated at room temperature for 20 min. Absorbance was read at a wavelength of 595 nm (Varian Inc., Palo Alto, CA). Samples were compared to a standard curve prepared using Bovine Serum Albumin as a standard (Pierce, Rockford, IL).

## Data Availability

All data generated or analyzed during this study are included in this published article or in prior published reports.

## Abbreviation

BNF: Biological nitrogen fixation
